# MiR-503 modulates epithelial-mesenchymal transition in silica-induced pulmonary fibrosis by targeting PI3K p85 and is sponged by lncRNA MALAT1

**DOI:** 10.1038/s41598-017-11904-8

**Published:** 2017-09-12

**Authors:** Weiwen Yan, Qiuyun Wu, Wenxi Yao, Yan Li, Yi Liu, Jiali Yuan, Ruhui Han, Jingjin Yang, Xiaoming Ji, Chunhui Ni

**Affiliations:** 1 Department of Occupational Medicine and Environmental Health, Key Laboratory of Modern Toxicology of Ministry of Education, School of Public Health, Nanjing Medical University, Nanjing, 211166 China; 2 Department of Hygiene, School of Public Health, Xuzhou Medical University, Xuzhou, 221004 China

## Abstract

Silicosis is a kind of chronic, progressive and incurable lung fibrotic diseases with largely unknown and complex pathogenesis and molecular mechanisms. Mounting evidence suggests that microRNAs (miRNAs, miRs) are involved in the pathogenesis of silicosis. Our previous study based on miRNA microarray had shown that the expression levels of miR-503 were down-regulated in mouse lung tissues of silica-induced pulmonary fibrosis. Here, we validated the decreased expression of miR-503 in the fibrotic mouse lung tissues, human bronchial epithelial cells (HBE) and human lung adenocarcinoma A549 cells which were exposed to silica. In addition, overexpressed miR-503 inhibited silica-induced pulmonary fibrosis by attenuating the severity and the distribution of lesions *in vivo* and limiting the process of epithelial-mesenchymal transition (EMT) *in vitro*. Our molecular study further demonstrated that PI3K p85 is one of the target genes of miR-503 and the downstream molecules (Akt, mTOR and Snail) are tightly associated with EMT. Furthermore, the up-regulated lncRNA Metastasis-associated lung adenocarcinoma transcript 1 (MALAT1), acted as a competing endogenous RNA (ceRNA), can directly bound to miR-503, which indicated that lncRNA MALAT1 may modulate the expression of miR-503 thus triggering the activation of downstream fibrotic signaling pathways. Taken together, our data suggested that MALAT1-miR-503-PI3K/Akt/mTOR/Snail pathway plays critical roles in silica-induced pulmonary fibrosis.

## Introduction

Silicosis, a kind of interstitial lung fibrotic disease, is incurable and irreversible, and usually caused by occupational exposure to silica dust^1, 2^. Alveolar epithelial cell injury and hyperplasia, persistent inflammation, extracellular matrix deposition and subsequent aberrant wound healing are common characteristics of silicosis^3^. In the process of pulmonary fibrosis, epithelial cells and macrophages are stimulated by the silica particles, secreting large amount of cytokines and inflammatory mediators, thus promoting epithelial cells transform to myofibroblasts through epithelial metaplasia, apoptosis, fibrocyte recruitment and EMT^4^. However, the molecular mechanisms underlying pulmonary fibrosis are still unclear.

Epithelial-mesenchymal transition (EMT) means a process that polar adjacent epithelial cells transform to non-polar mesenchymal cells which lack cell-cell contacts and increase cell mobility^5^. EMT plays an important role in the development of pulmonary fibrosis and has been proved to be a valuable incident which occurs in the alveolar type II epithelial cells^6^. Myofibroblasts accumulate and secrete large amount of collagen during the formation of fibrosis, which lead to the failure of lung function. Studies have shown that pulmonary fibrosis is a process undergoing the activation of interstitial fibroblasts that convert to myofibroblasts to form the fibrotic collagen network^7^. Moreover, a population of the fibroblasts involved in the fibrotic process is thought to originate from the transition of the epithelial cells through the process of EMT^8^.

MicroRNAs (miRNAs, miRs) are a class of ~22-nucleotide-long small non-coding RNAs, which regulate the genes expression by pairing to the 3′UTR of the genes, thus repressing translation or degradation of target mRNAs^9^. Each miRNAs could bind to several different target mRNAs leading to play numerous biological functions^10^. Moreover, accumulating evidence indicates that the aberrant expression of miRNAs play an important role in lung fibrosis, such as myofibroblast differentiation and EMT^11^. *Liu et al*. demonstrated that the down-regulation of miR-21 alleviates the pro-fibrotic activity of TGF-β1 in fibroblasts in IPF^12^. Alternatively, miRNAs can directly participate in EMT in pulmonary epithelial cells. *Antognelli et al*. have identified that miR-21 plays positive regulatory role in min-U-Sil 5 crystalline silica (MS5)-induced EMT in BEAS-2B cells^13^. *Huleihel L et al*. confirmed that miRNA let-7d attenuates EMT process in lung fibrosis^14^. To confirm the therapeutic importance of miRNAs in silica-induced pulmonary fibrosis, our previous work have revealed that miR-486-5p and miR-489 play important anti-fibrotic roles in silica-induced pulmonary fibrosis^15, 16^.

miR-503, located on the chromosome Xq26.3, is an intragenic miRNA, and belongs to the miR-16 family^17^. In addition, miR-503 has been reported to exert diverse biological functions in several kinds of cancer, such as hepatocellular carcinoma (HCC), cervical cancer, prostate cancer, etc. refs 18–21. For example, *Long et al*. reported that miR-503 inhibits cell proliferation in human breast cancer^22^. *Zhou et al*. discussed the role of miR-503 in tumor angiogenesis and growth^23^. *Peng et al*. identified that miR-503 inhibits cell growth and EMT in gastric cancer^24^. However, fewer studies have concentrated on the regulation of miR-503 in the pathological process of lung fibrosis, particularly silicosis. Our previous miRNA microarray data have shown that the expression of miR-503 is decreased in mouse lung tissues of the silica group compared to the control group. The results indicated that miR-503 might be a potentially vital miRNA for the therapy of silicosis.

Long non-coding RNAs (lncRNAs) are non-protein coding transcripts longer than ~200 nucleotides and have drawn much attention recently^25^. Emerging evidence has confirmed that several lncRNAs play pivotal roles in the regulation of various biological processes, such as cell proliferation and differentiation^26, 27^, metastasis^28^ and EMT^29, 30^. And the most acknowledged molecule mechanism of lncRNAs is functioning as “sponges” of miRNAs, modulating the activity of miRNAs. Therefore, lncRNAs are also known as competing endogenous RNAs (ceRNAs)^31^. It is increasingly understood that lncRNAs are important molecules involving in various diseases, especially cancers^32, 33^. And only a few reports have identified the relationship between lncRNAs and pulmonary fibrosis. *Sun et al*. have demonstrated that two lncRNAs uc.77 and 2700086A05Rik regulate EMT in the mouse model of pulmonary fibrosis^34^.

Metastasis-associated lung adenocarcinoma transcript 1 (MALAT1), also known as NEAT2, is a widely and highly expressed single-exon lncRNA, which was originally identified in non-small cell lung cancers^35^. MALAT1 can regulate several biological processes through acting as a ceRNA. A previous study reported that MALAT1 modulates radiosensitivity of HR-HPV+ cervical cancer via sponging miR-145^36^. Our previous data revealed that the relative expression of miR-503 is reduced in the lung tissues of the silica group compared to the control group by microarray analysis. And in the present study, the roles of miR-503 and the relationship between lncRNA MALAT1 and miR-503 in the silica-induced EMT were investigated. Our results indicate new fibrotic mechanisms and provide potential therapeutic approaches for silica-induced pulmonary fibrosis.

## Results

### The expression of miR-503 is down-regulated in the lung tissues of mice with silica-induced pulmonary fibrosis

**Figure 1:**
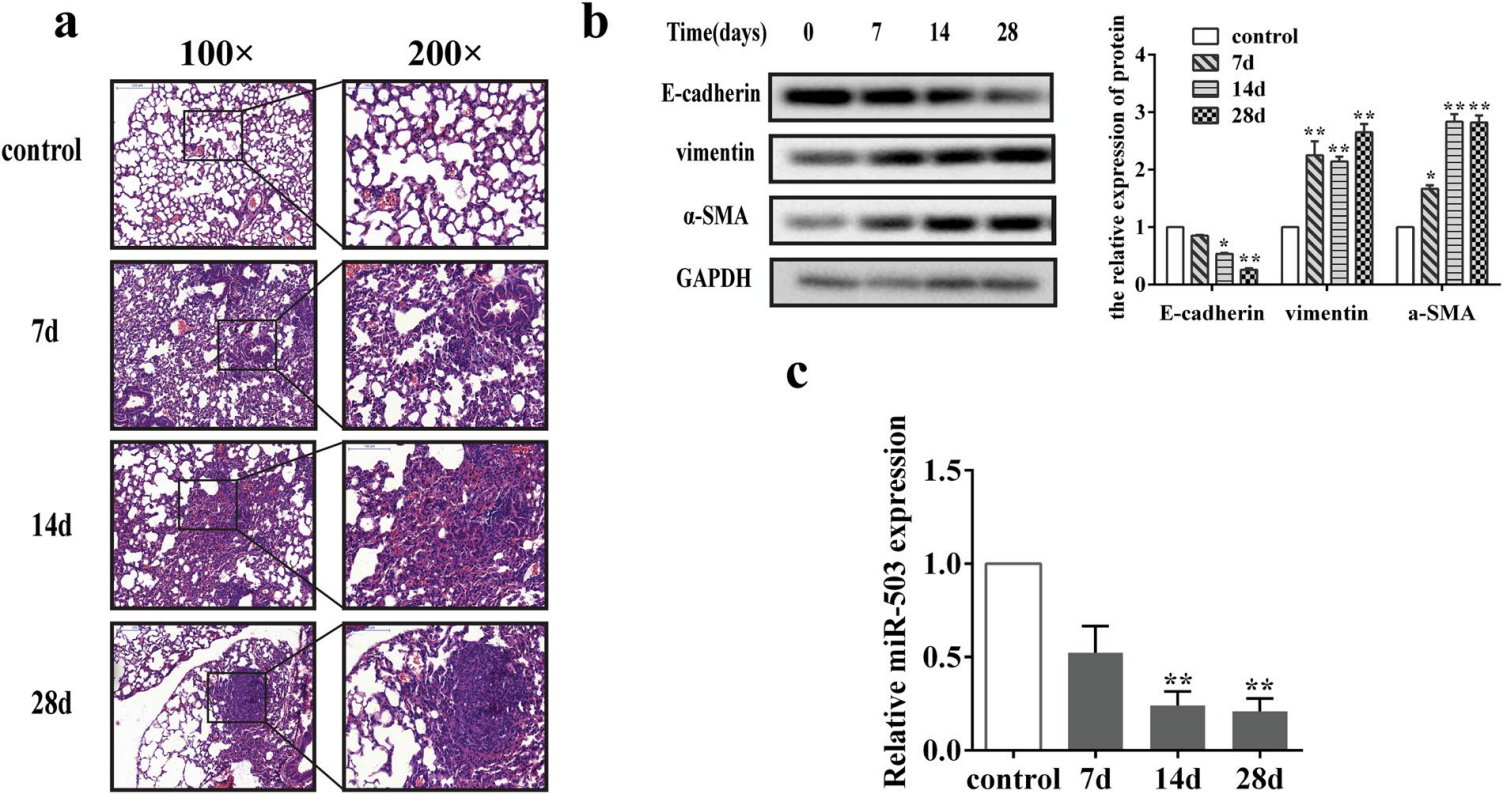
The expression of miR-503 is decreased in mouse lung tissues of silica-induced pulmonary fibrosis. (**a**) The sections were stained with hematoxylin and eosin as described in the materials and methods, which present the typical pathological alterations of pulmonary fibrosis. (**b**) Proteins of lung tissues were extracted and protein levels of EMT markers (E-cadherin, vimentin and α-SMA) were determined by western blot. Relative protein levels (means ± SD, n = 3) were analyzed by ImageJ, *P < 0.05 and **P < 0.01 versus the control group. (**c**) Total RNA of mouse lung tissues were extracted and relative miR-503 expression levels were examined by qRT-PCR, U6 was used as the interval reference, **P < 0.01 versus the control group.

To investigate the pathogenesis of silicosis, we developed a mouse model of pulmonary fibrosis by intratracheal administration of silica suspension as previously described. The pathological changes displayed increasing severity of lung fibrosis in a time-dependent manner. And on day 28 after silica instillation, the typical fibrotic nodules changes were observed by hematoxylin and eosin (H&E) staining analysis (Fig. 1a). To determine whether EMT occur in this model, the protein expression levels of epithelial marker (E-cadherin) and mesenchymal markers (vimentin and α-SMA) were examined in mice lung tissues. Western blot analysis revealed that the E-cadherin protein level was decreased, while the protein levels of vimentin and α-SMA were increased along with the extension of the treatment time (Fig. 1b). The results above indicated that we have successfully established the silica-induced mouse pulmonary fibrosis model. Previous microarray study of mouse lung fibrosis showed that the expression of miR-503 on day 28 after silica treatment was decreased about three-fold changes compared with the control group (Supplementary Fig. 1a). To validate the expression level of miR-503 in this re-established model, qRT-PCR analysis was performed and displayed markedly decreased expression of miR-503, about five-fold changes, in the day 28 group as compared with the control group (Fig. 1c). All these results indicated that miR-503 is significantly down-regulated in the fibrotic mice lung tissues, but whether miR-503 influences the pathological process and development of the pulmonary fibrosis needs further investigation.

### Increased miR-503 attenuates the EMT *in vivo*

**Figure 2:**
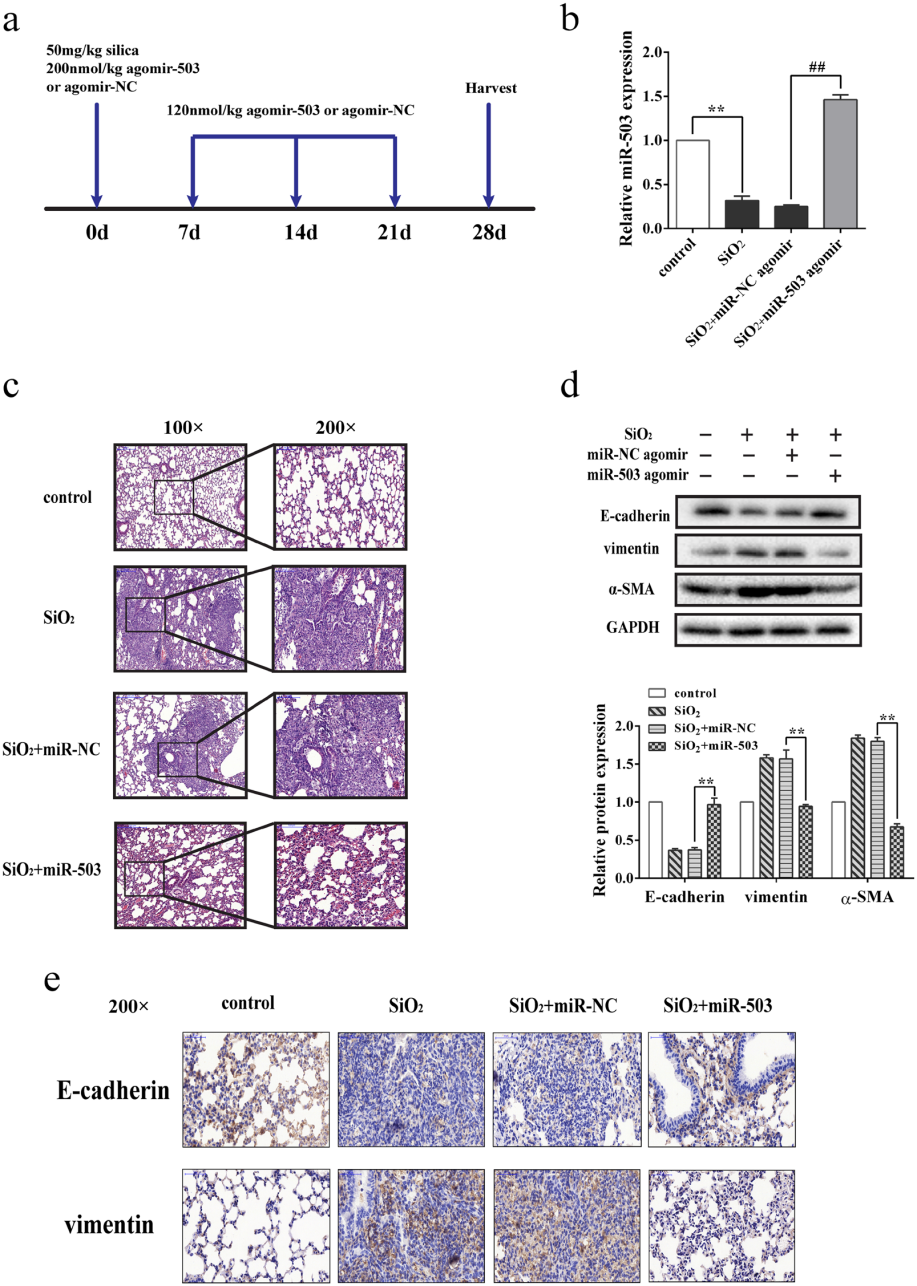
Increased miR-503 attenuates EMT *in vivo*. (**a**) The mouse model of miR-503 overexpression in silica-induced mouse pulmonary fibrosis. The C57BL/6 mice were co-transfected 200 nmol/kg either miR-503 or miR-NC agomir with 50 mg/kg silica suspension via intratracheal instillation, and the mice were injected with 120 nmol/kg miR-503 or miR-NC agomir via the tail vein on day 7, 14 and 21. Then the tissues were harvested on day 28 (n = 8 for each group). (**b**) The expression level of miR-503 was increased in the lung tissues treated with miR-503 agomir compared with the group treated with SiO_2_ + miR-NC agomir. **P < 0.01 versus the control group, and ^##^ *P* < 0.01 versus the SiO_2_ + miR-NC agomir group. (**c**) Sections stained with hematoxylin and eosin reflected that the severity of the lung fibrosis of the group treated with miR-503 agomir was not as serious as the group treated with SiO_2_ or SiO_2_ + miR-NC agomir. (**d**) Protein levels of the EMT markers were significantly reversed compared with the SiO_2_ + miR-NC agomir group when injected with miR-503 agomir. Relative protein levels (means ± SD, n = 3) were analyzed by ImageJ, and were determined by normalization with the interval reference GAPDH, **P < 0.01 versus the SiO_2_ + miR-NC agomir group. (**e**) Immunohistochemistry assays were performed to measure the expression of E-cadherin and vimentin (200× magnification).

Having verified the expression of miR-503 was down-regulated in the mouse lung tissues of silica-induced pulmonary fibrosis, then we explored whether raising the expression of miR-503 *in vivo* alleviates the process of EMT and influences the pathological process of silicosis. To confirm our assumption, the model was conducted by dripping miR-503 agomir or miR-NC intratracheally following the instillation of silica at day 0, and then miR-503 agomir or miR-NC was injected via the tail vein on day 7, 14, and 21 after silica treatment. The mice were sacrificed at day 28 and the lungs were harvested (Fig. 2a). The results showed down-regulation of miR-503 was found in the silica group compared with the control group, but elevated when treated with miR-503 agomir (Fig. 2b). And pathological analysis indicated that miR-503 agomir really relieved the severity and distribution of lung lesions compared with the silica plus miR-NC group (Fig. 2c and Table 1). Consistently, the up-regulation of miR-503 increased the protein expression level of E-cadherin and decreased the expression levels of vimentin and α-SMA, thus alleviating the process of EMT (Fig. 2d). It was also proved that miR-503 overexpression increased E-cadherin expression and reduced vimentin in fibrotic lung tissues by using immunohistochemistry assays (Fig. 2e). Taken together, these results indicated that miR-503 is able to alleviate the development and pathological process of mouse pulmonary fibrosis *in vivo* via the EMT-suppressive effects.

**Table 1:** Effect of miR-503 agomir on the severity and distribution of the mouse lung fibrosis.

Groups	Lesion severity grade						Average severity grade	Lesion distribution grade						Average severity grade
	0	1	2	3	4	5		0	1	2	3	4	5	
Control	8						—	8						—
SiO_2_		2	1	1	3	1	3.00 ± 1.51^*^		1		5	2		3.00 ± 0.93^*^
SiO_2_ + miR-NC		1	2	3	2		2.75 ± 1.04^*^		1	1	4	2		2.88 ± 0.99^*^
SiO_2_ + miR-503	1	3	3	1			1.50 ± 0.93^*#^	1	2	3	2			1.75 ± 1.04^*#^

*P < 0.05 versus the control group, ^#^ *P* < 0.05 versus the group treated with silica plus miR-NC (independent samples *t* test).

### MiR-503 blocks the process of EMT via targeting PI3K p85

**Figure 3:**
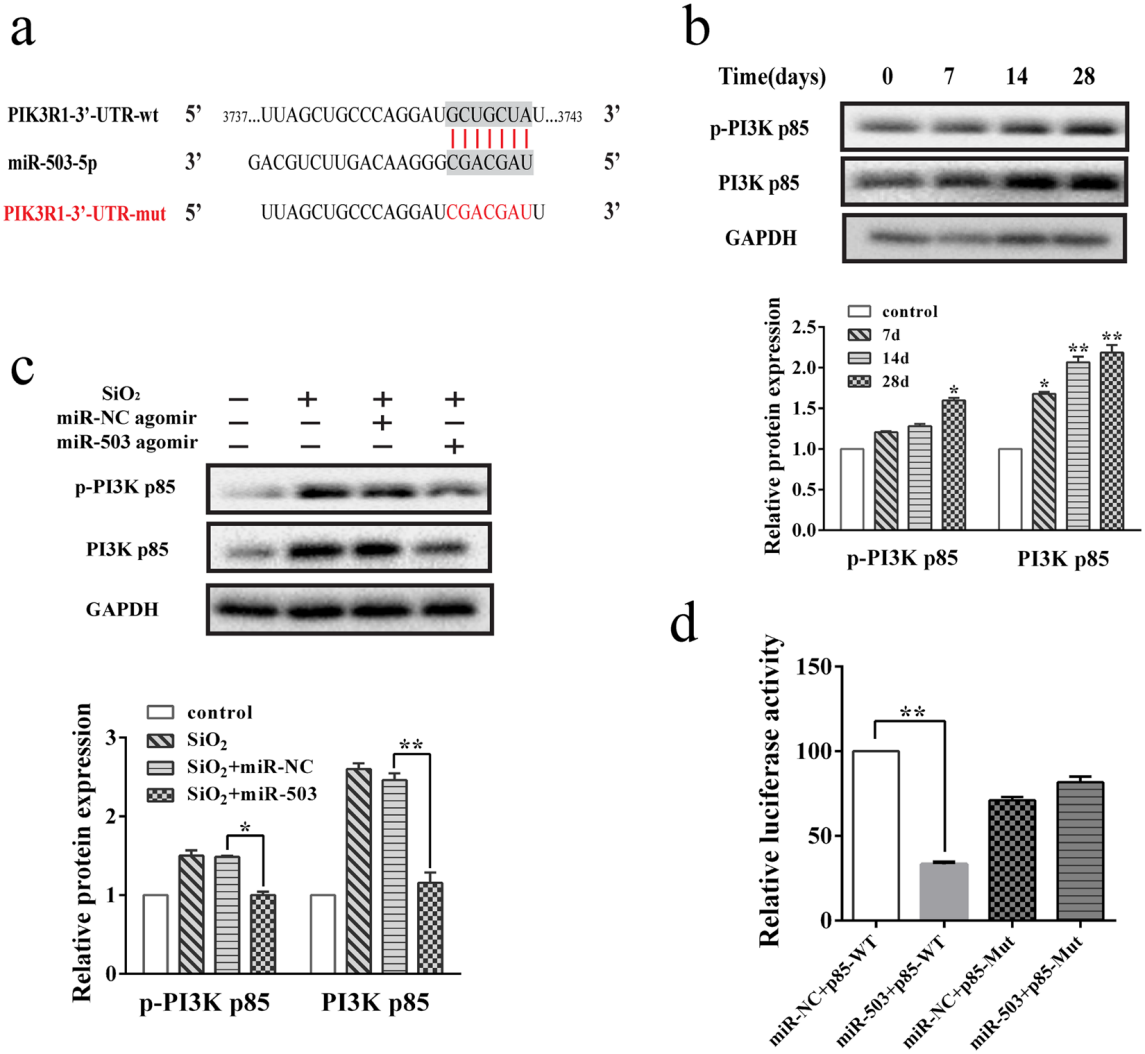
MiR-503 directly targets and down-regulates expression of PI3K p85. (**a**) Schematic diagram showing miR-503 targeting sequences or mutated sequences from the 3′-UTR of the PI3K p85 gene. (**b**) The protein levels of p-PI3K p85 and PI3K p85 in mouse lung tissues treated with silica for 7, 14 and 28 days were detected by western blot analysis. And the relative protein levels (means ± SD, n = 3) were analyzed by ImageJ, *P < 0.05 and **P < 0.01 versus the control group. (**c**) The protein expression of PI3K p85 in mouse lung tissues treated with miR-503 agomir for 28 days were determined by western blot and found decreased compared with the SiO_2_ + miR-NC agomir group, *P < 0.05 and **P < 0.01 versus the SiO_2_ + miR-NC agomir group. (**d**) Effects of miR-503 mimic on PI3K p85 3′-UTR luciferase reporters in HBE cells. Luciferase activities were calculated as the ratio of firefly/renilla activities and normalized to the miR-NC + p85 WT group, **P < 0.01 versus the miR-NC + p85 WT group.

From the study above, we can easily come to the conclusion that miR-503 plays a pivotal role in the process of EMT, thus limiting mouse lung fibrosis. But the molecular mechanisms underlying the EMT-suppressive effects of miR-503 are still unclear. Previous studies have indicated that microRNAs play its biological functions by binding to the 3′UTR of the genes, thus silencing the gene expression correlated with several complex gene networks. Therefore, we use TargetScan bioinformatics software to predict the target genes of miR-503. It was found that PI3K p85 might be the functional potential target of miR-503 (Fig. 3a). Moreover, *Yang et al*.^27^ reported that PI3K p85 is a direct target of miR-503 in non-small cell lung cancer. So we then detected the protein levels of PI3K p85 in mouse lung tissues by Western blot. The results showed that the expression levels of p-PI3K p85 (activated PI3K p85) and PI3K p85 significantly increased in mouse lung tissues after silica treatment (Fig. 3b). And up-regulated miR-503 in a mouse model also reduced the protein levels of p-PI3K p85 and PI3K p85 (Fig. 3c). To verify whether miR-503 is capable of regulating PI3K p85 via the binding sites in its 3′-UTR, we constructed the 3′-UTR containing the predicted miR-503 binding site downstream of the firefly luciferase coding region in the psi-CHECK2-REPORT luciferase vector. The luciferase reporter vectors together with the miR-503 mimic or miRNA mimic control were transfected into the HBE and A549 cells. For the wild type with PI3K p85 reporter, over-expression of miR-503 significantly reduced its relative luciferase activity compared to group transfected with non-target miRNA mimic control, whereas this effect was abolished in the case of the mutant reporter in which the miR-503 binding site was mutated (Fig. 3d and Supplementary Fig. 1b). All these results strongly suggest that PI3K p85 can be targeted by miR-503 directly.

**Figure 4:**
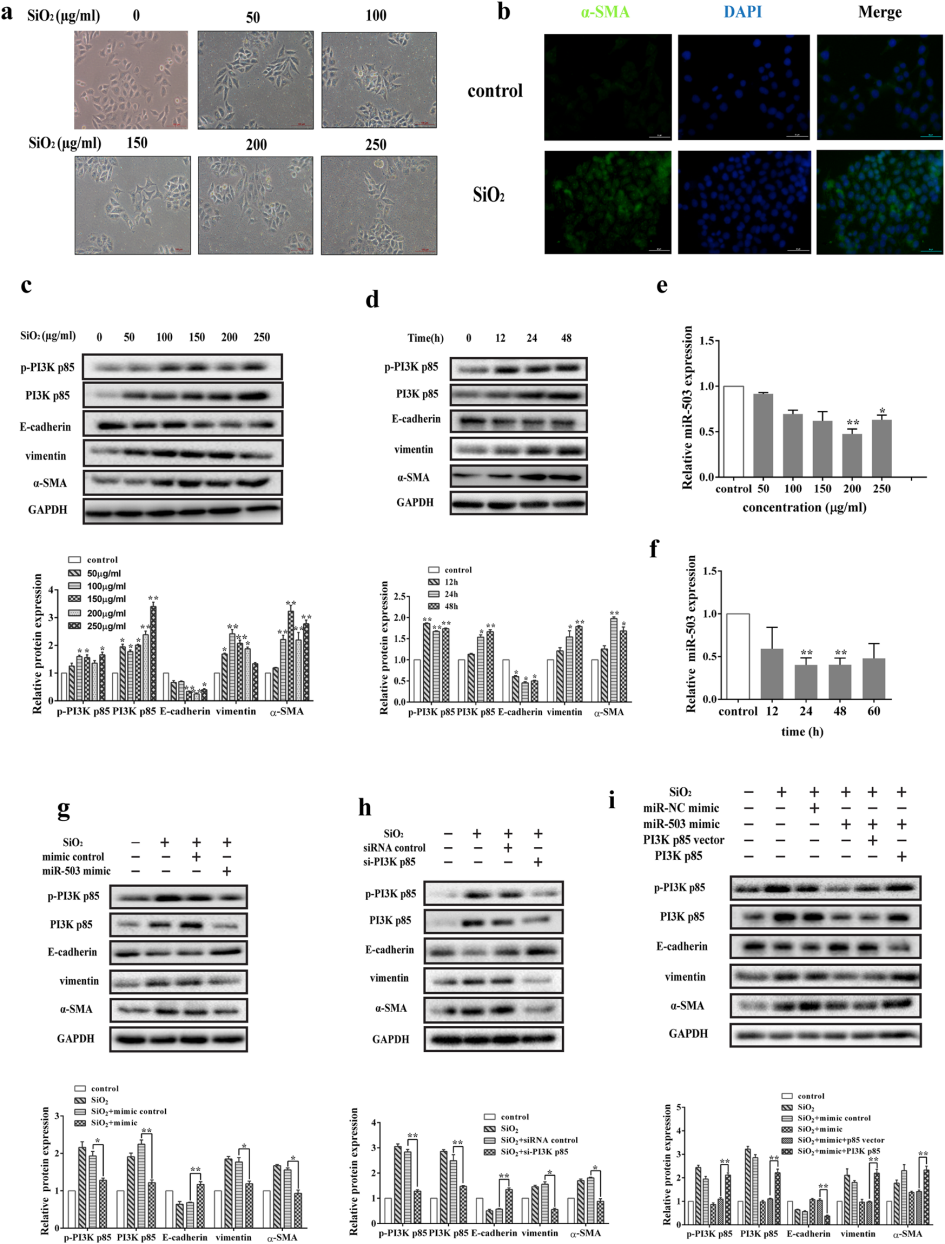
MiR-503 blocks the process of EMT via targeting PI3K p85 in HBE cells. (**a**) The cells (HBE) morphology shifted toward mesenchymal-like phenotype when treated with different concentrations of silica suspension (0, 50, 100, 150, 200, 250 μg/ml) for 48 h. The pictures were captured by the inverted microscope (Olympus). The scale bar is 100 μm. (**b**) The fluorescence intensity of α-SMA in HBE cells treated with 200 μg/ml silica was notably higher than the control group. The scale bar is 50 μm. (**c**,**d**) The protein expression levels of p-PI3K p85, PI3K p85, vimentin and α-SMA were obviously elevated and the E-cadherin protein levels were decreased when treated with different doses of silica for 48 h and 200 μg/ml silica for different time points. And the relative protein expression levels were analyzed by the ImageJ program. *P < 0.05 and **P < 0.01 versus the control group. (**e**,**f**) The miR-503 expression levels were significantly decreased in HBE cells treated with different doses of silica for 48 h and 200 μg/ml silica for different time points, *P < 0.05 and **P < 0.01 versus the control group. (**g**) MiR-503 mimics were transfected to HBE cells one day before treated with silica and then cultured for 48 h. MiR-503 mimics reversed the protein expression levels of the target PI3K p85 and EMT markers (E-cadherin, vimentin, α-SMA) in HBE cells, *P < 0.05 and **P < 0.01 versus the SiO_2_ + miR-NC mimic group. (**h**) The siRNA of PI3K p85 reduced the protein expression of PI3K p85 and alleviated the process of epithelial mesenchymal transformation, *P < 0.05 and **P < 0.01 versus the SiO_2_ + siRNA control group. (**i**) Overexpression of PI3K p85 significantly counteracted the inhibitory effects of miR-503 in the process of EMT by rescue experiment, **P < 0.01 versus the SiO_2_ + mimic + p85 vector group.

Since the cell lines will be used to investigate the further molecular mechanisms of miR-503 in silica-induced pulmonary fibrosis, we need to prove that EMT is one of important events when epithelial cells are treated with silica. we treated two cell lines (HBE and A549) and murine primary alveolar type II epithelial cells (AECs) with different concentrations of silica. HBE cells displayed a morphological shift from a rounded or cobblestone-stone shaped, epithelial-like morphology to a spindle-shaped, star-like mesenchymal morphology (Fig. 4a) and consistent with an up-regulation of the fluorescence intensity of α-SMA compared with the control group (Fig. 4b). A549 cells became thinner and transited from epithelial state to mesenchymal state when treated with silica (Supplementary Fig. 1c). Meanwhile, the protein expression levels of vimentin and α-SMA were also obviously elevated and E-cadherin decreased when treated with different doses of silica for 48 h and 200 μg/ml (150 μg/ml for A549 cells) silica at the different time points in HBE and A549 cells (Fig. 4c and d; Supplementary Fig. 1d and e). Murine primary AECs were treated with different doses of silica (0, 100, 150 and 200 μg/ml) for 48 h, the cells underwent morphological conversions toward the mesenchymal-like cells(Supplementary Fig. 1f), and the results of western blot also showed that EMT occurred (Supplementary Fig. 1g). These results demonstrated that silica treatment could induce EMT in those epithelial cells. As the isolation and culture of Murine primary AECs are complex and the cell passage is limited, A549 cell line, adenocarcinomic human alveolar basal epithelial cells possessed the characteristics of AECs, has been used in many studies for the replacement of AECs. Therefore, we use the HBE and A549 cell lines to explore the further molecular mechanisms in present study.

In silica-treated HBE and A549 cell lines, the protein expression levels of p-PI3K p85 and PI3K p85 increased (Fig. 4c and d and Supplementary Fig. 1d and e), and the miR-503 levels were decreased significantly (Fig. 4e and f and Supplementary Fig. 1h and i), which showed the inverse correlation with PI3K p85 expressions. In addition, we confirmed that ectopic expression of miR-503 via mimic transfection could partly reverse the morphological changes of HBE cells (Supplementary Fig. 1j) and suppressed the expression of p-PI3K p85, PI3K p85, vimentin and α-SMA, and correspondingly enhanced the expression of E-cadherin at the protein level (Fig. 4g and Supplementary Fig. 1k). Similarly, knockdown of PI3K p85 by siRNA resulted in decreased vimentin and α-SMA expression, and increased E-cadherin expression (Fig. 4h and Supplementary Fig. 1l). The correlation between miR-503 and PI3K p85 was further determined by the rescue experiment in both cell lines. We overexpressed PI3K p85 by the co-transfection of pcDNA3.1-PI3K p85 plasmid with miR-503 mimic. Interestingly, overexpression of PI3K p85 largely counteracted the inhibitory effects of miR-503 mimic (Fig. 4i and Supplementary Fig. 1m). Taken together, our results indicated that miR-503 alleviates the process of EMT by down-regulating the expression of PI3K p85.

### MiR-503 influences EMT through PI3K/Akt/mTOR/Snail signaling pathway

**Figure 5:**
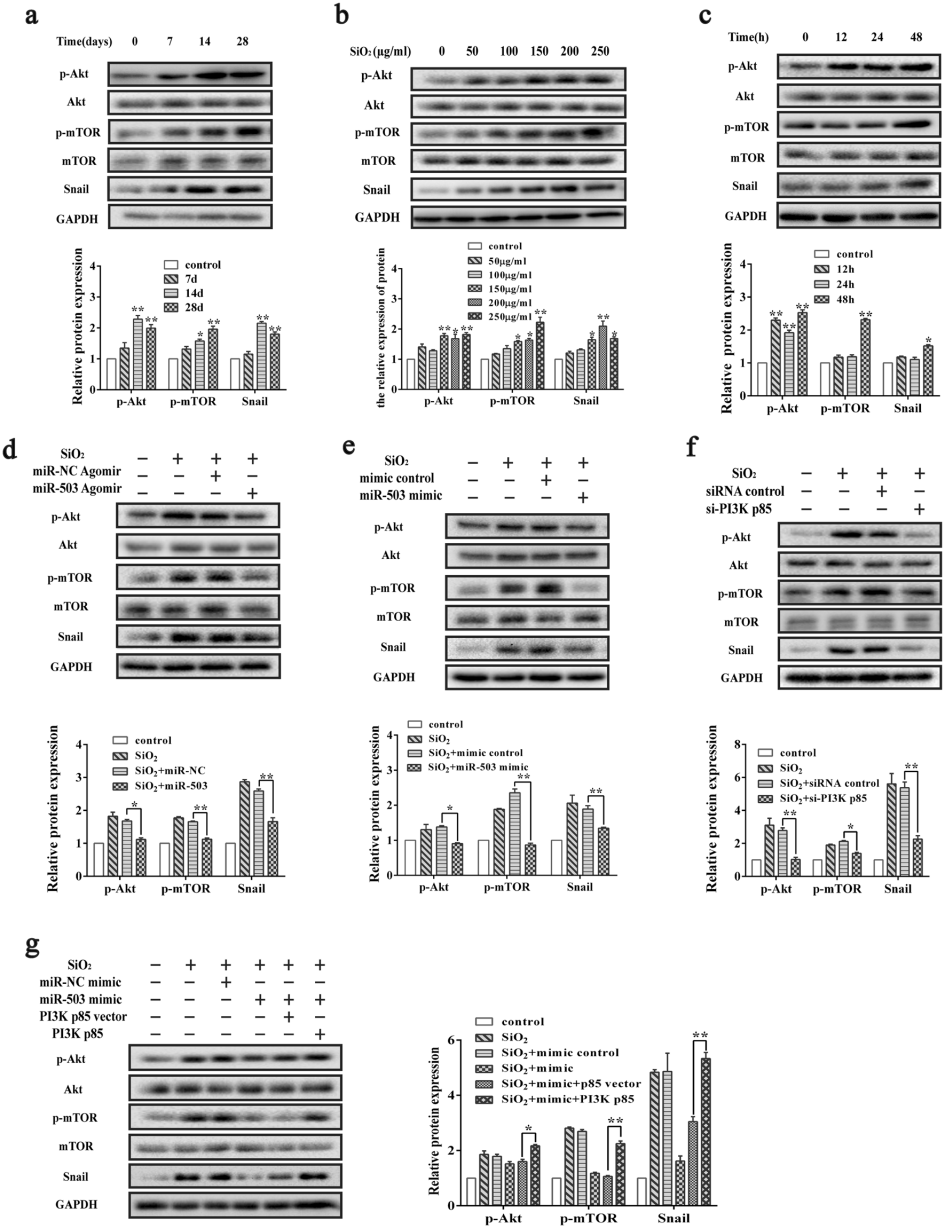
MiR-503 influences EMT through PI3K/Akt/mTOR/Snail signaling pathway in HBE cells. (**a**) Western blot analysis was performed to detect the protein expression levels of Akt, phosphorylated-Akt, mTOR, phosphorylated-mTOR and Snail in mouse lung tissues. And the immunosignals were quantified by using densitometric scanning software ImageJ. The relative protein levels of p-Akt were normalized with total levels of Akt, and the relative protein levels of p-mTOR were normalized with total levels of mTOR, *P < 0.05 and **P < 0.01 versus the control group. (**b**,**c**) The protein expression levels of p-Akt, p-mTOR and Snail in HBE cells were gradually increased when treated with different concentrations of silica for 48 h and 200 μg/ml silica for different time points, *P < 0.05 and **P < 0.01 versus the control group. (**d**) The miR-503 agomir were tail-injected into the mice for 28 days and decreased the protein expression levels of p-Akt, p-mTOR and Snail in mouse lung tissues of the model, *P < 0.05 and **P < 0.01 versus the SiO_2_ + miR-NC agomir group. (**e**) miR-503 mimics were transfected to HBE cells for 48 hours and significantly decreased the protein levels of p-Akt, p-mTOR and Snail, *P < 0.05 and **P < 0.01 versus the SiO_2_ + miR-NC mimic group. (**f**) The transfection of PI3K p85 siRNA together with the silica treatment for 48 hours significantly reduced the expression of p-Akt, p-mTOR and Snail, *P < 0.05 and **P < 0.01 versus the SiO_2_ + siRNA control group. (**g**) Co-transfection with PI3K p85 overexpression plasmid and miR-503 mimics for 48 hours restored the protein expression levels of p-Akt, p-mTOR and Snail which were inhibited by miR-503 mimic, *P < 0.05 and **P < 0.01 versus the SiO_2_ + mimic + p85 vector group.

The results obtained here have preliminarily illustrated the target gene of miR-503, while the downstream molecular signaling mechanisms need further research. Akt and mTOR are two molecules downstream to PI3K, and Snail is a key transcription factor which were reported to modulate the expression of EMT markers and induce EMT^37^. Therefore, we examined whether miR-503 could inhibit EMT through PI3K/Akt/mTOR/Snail signaling pathway. Western analysis of the silica-induced mouse lung fibrosis revealed that the variation trend of p-Akt, p-mTOR and Snail protein expression is similar with PI3K p85 (Fig. 5a). Consistently, in HBE cells, the protein expression levels of p-Akt, p-mTOR and Snail were gradually increased when treated with different concentrations of silica for 48 h and 200 μg/ml silica for different time points (Fig. 5b and c). On the contrary, overexpression of miR-503 repressed the protein expression of p-Akt, p-mTOR and Snail *in vivo* (Fig. 5d) and *in vitro* (Fig. 5e and Supplementary Fig. 2a). Silencing the expression of PI3K p85 also significantly attenuated the protein expression levels of downstream p-Akt, p-mTOR and Snail (Fig. 5f and Supplementary Fig. 2b). Our rescue experiment also showed that co-transfection with pcDNA3.1-PI3K p85 and miR-503 mimic restored the protein expression levels of p-Akt, p-mTOR and Snail which were inhibited by miR-503 mimic (Fig. 5g and Supplementary Fig. 2c). Based on these findings, we concluded that miR-503 suppresses EMT by targeting PI3K/Akt/mTOR/Snail pathway in silica-induced pulmonary fibrosis.

### LncRNA MALAT1 promotes EMT via binding to miR-503 directly

**Figure 6:**
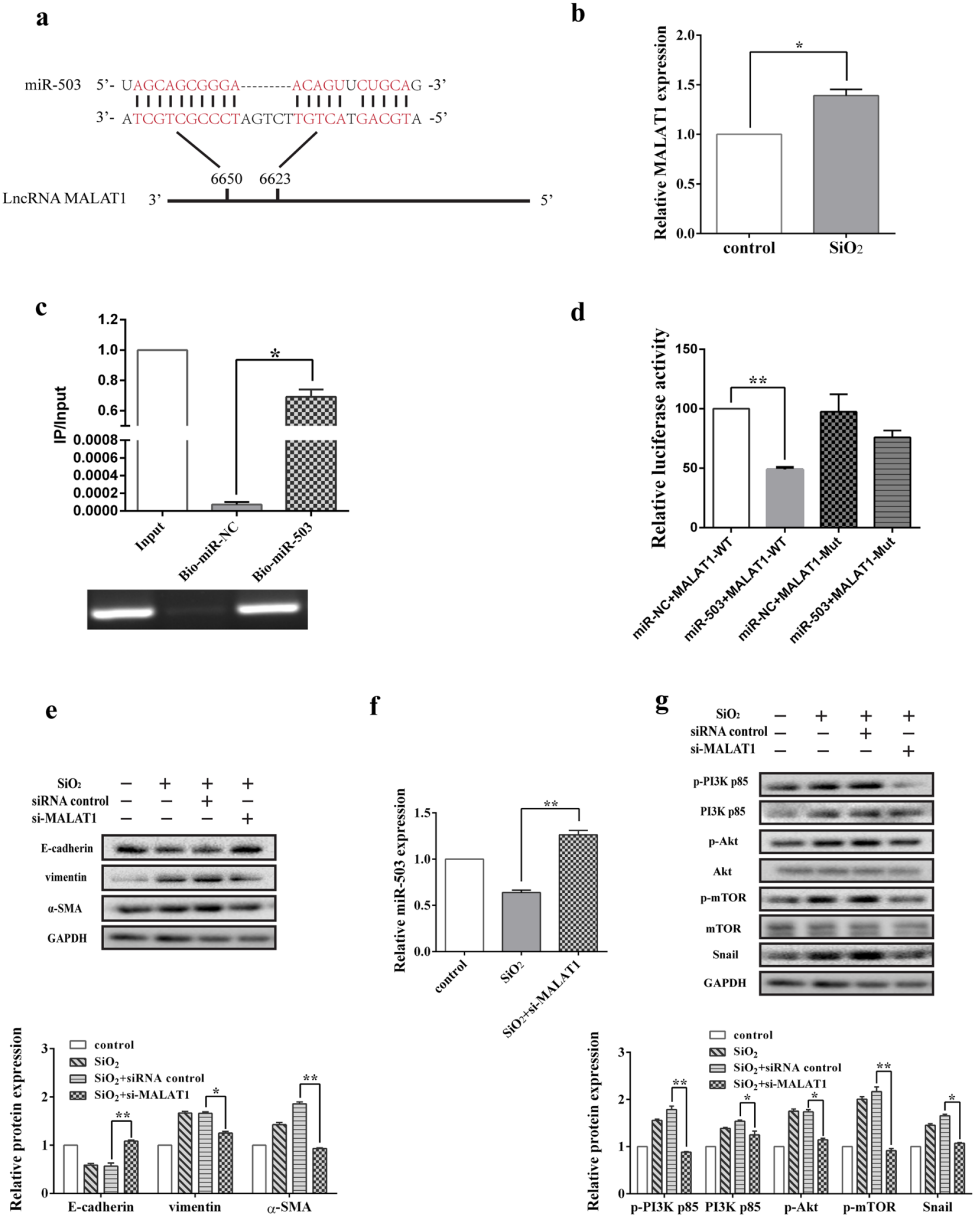
LncRNA MALAT1 promotes EMT via binding to miR-503 directly. (**a**) Predicted binding sites for miR-503 in MALAT1. (**b**) The relative expression level of lncRNA MALAT1 was significantly increased in HBE cells after treated with 200 μg/ml silica for 48 h compared to the control group, *P < 0.05 versus the control group. (**c**) MALAT1 is associated with miR-503. Biotinylated miR-503 (bio-miR-503) and miR-NC were incubated with the extracted RNA of HBE cells (10 μl of the RNA samples were reserved for input) to pull down lncRNA MALAT1. After the biotin-labeled pull-down assay, MALAT1 expression levels were analyzed by qRT-PCR and RT-PCR, *P < 0.05 versus the Bio-miR-NC group. (**d**) The luciferase reporter gene assay was performed to identify the interaction between lncRNA MALAT1 and miR-503 in HBE cells. Luciferase activities were calculated as the ratio of firefly/renilla activities and normalized to the miR-NC + MALAT1 WT group, **P < 0.01 versus the miR-NC + MALAT1 WT group. (**e**) LncRNA MALAT1 siRNA was transfected to HBE cells and significantly alleviated the process of EMT assessed by Western blot, *P < 0.05 and **P < 0.01 versus the SiO_2_ + siRNA control group. (**f**) The level of miR-503 in HBE cells was increased after silencing of MALAT1 determined by qRT-PCR, **P < 0.01 versus the SiO_2_ group. (**g**) The protein levels of p-PI3K p85, PI3K p85, p-Akt, p-mTOR and Snail were repressed with the treatment of si-MALAT1, *P < 0.05 and **P < 0.01 versus the SiO_2_ + siRNA control group.

Recently, several reports have confirmed that lncRNAs may act as competing endogenous RNAs (ceRNAs) or molecular sponges of miRNAs, modulating their expression and biological functions. The aforementioned studies have shown that the expression of miR-503 is down-regulated in the silica-treated HBE cells and A549 cells (Fig. 4e,f and Supplementary Fig. 1d,e), thus we wonder whether the expression of miR-503 is regulated by lncRNA. The predict software (Starbase v2.0 and RegRNA 2.0) was used to identify the potential target lncRNA of miR-503 and found a putative complementary sequence for miR-503 in lncRNA MALAT1 at position 6623–6650 (Fig. 6a). To further investigate whether MALAT1 is a functional target of miR-503, the relative expression of lncRNA MALAT1 in silica-treated HBE cells was detected by qRT-PCR. The results showed that the expression of lncRNA MALAT1 was significantly up-regulated in the silica-treated group compared to the control one (Fig. 6b), which is negatively correlated with miR-503 expression. Furthermore, RNA pull-down assay was applied to validate whether miR-503 could bind to lncRNA MALAT1. It was found that around 70% of MALAT1 was pulled down by biotin-labeled miR-503 compared with the input in HBE cells or the negative control with a biotin-labeled miR-NC (irrelevant sequence) (Fig. 6c). To further investigate whether miR-503 target the predicted binding sites in lncRNA MALAT1, we subsequently performed the dual luciferase reporter gene assay in HBE and A549 cells. A significant decrease in relative luciferase activity was observed when the pGL3-MALAT1-wt-3′-UTR vector was co-transfected with the miR-503 mimic but not with the miRNA mimic control (Fig. 6d and Supplementary Fig. 3a). Taken together, it appears that lncRNA MALAT1 is able to bind to miR-503 directly.

We then examined whether lncRNA MALAT1 is correlated with the process of EMT in silica-induced pulmonary fibrosis. We found that knockdown of lncRNA MALAT1 together with the treatment of silica could obviously attenuate the process of EMT in HBE and A549 cells (Fig. 6e and Supplementary Fig. 3b). After we have confirmed lncRNA MALAT1 could act as a sponge of miR-503, and miR-503 could influence EMT through PI3K/Akt/mTOR/Snail signaling pathway, it is need to test whether lncRNA MALAT1 regulates the process of EMT via the miR-503-PI3K/Akt/mTOR/Snail signaling pathway. The results revealed that inhibition of lncRNA MALAT1 could significantly increase the expression of miR-503 in these two cell lines (Fig. 6f and Supplementary Fig. 3c). By contrast, the protein expression of p-PI3K p85, PI3K p85 and its downstream molecules p-Akt, p-mTOR and Snail were down regulated after silencing the expression of lncRNA MALAT1 (Fig. 6g and Supplementary Fig. 3d). These data suggest that lncRNA MALAT1 could affect the process of EMT in silica-induced pulmonary fibrosis via miR-503-PI3K/Akt/mTOR/Snail signaling pathway.

## Discussion

**Figure 7:**
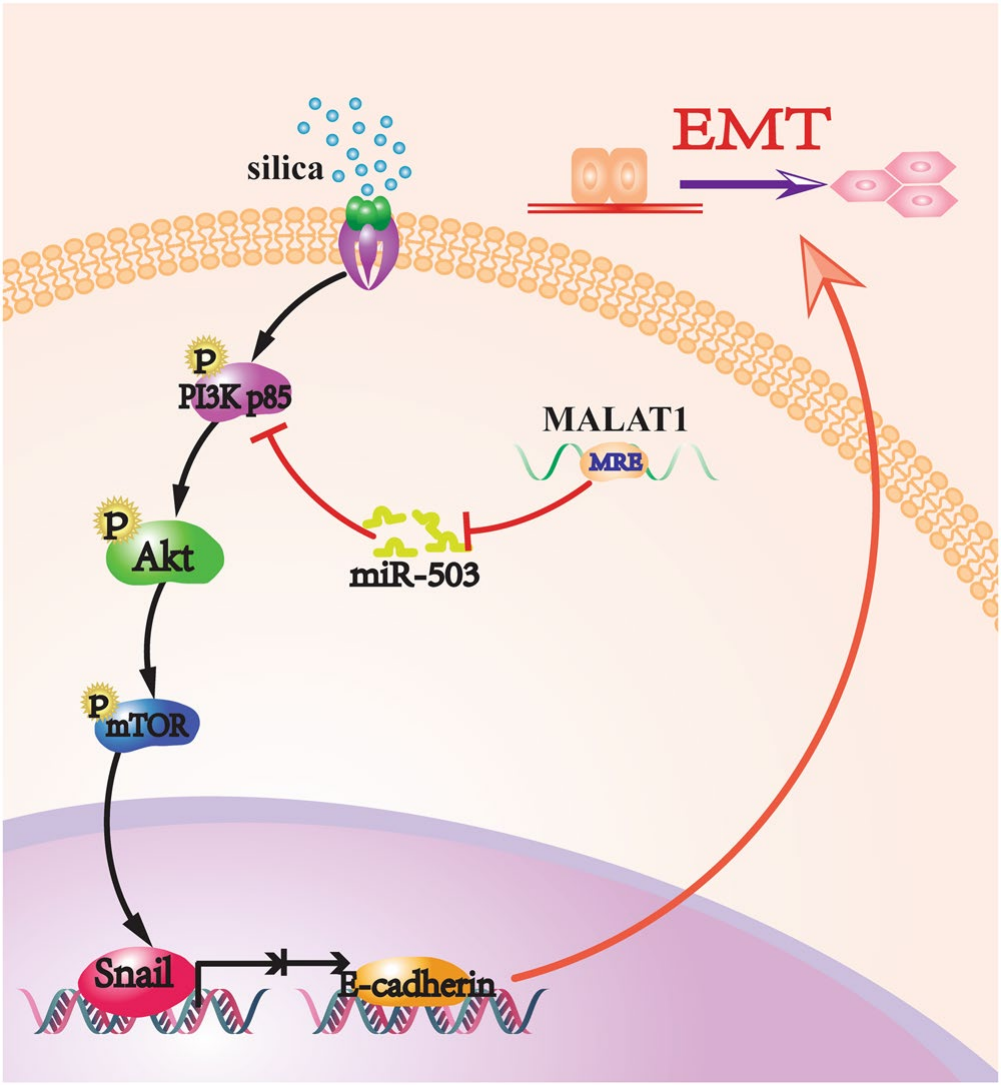
The signaling pathway for miR-503 playing its EMT-suppressive role in silica-induced pulmonary fibrosis. Silica treatment resulted in the enhanced expression of lncRNA MALAT1, which competitively binding to miR-503 and depressed its expression. When miR-503 was silenced, PI3K p85 binding to miR-503 was released and thereby activated the downstream molecules, thus causing intensified process of EMT.

Silicosis is a chronic progressive lung fibrotic disease. At present, there are no effective treatments for silicosis since the pathogenesis is not uncovered. Accumulated studies revealed deregulated miRNAs in several biological functions^38–40^, including the process of pulmonary fibrosis^12, 14, 15^. Our previous microarray analysis showed miR-503 is down-regulated in the lung fibrotic tissue which suggested miR-503 may play an important role in the process of pulmonary fibrosis. Based on our findings, a functional model was proposed to integrate miR-503 with downstream PI3K/Akt/mTOR/Snail signaling and upstream endogenous ‘sponge’ lncRNA MALAT1 regulation network (Fig. 7). MiR-503 binds to the 3′-UTR region of PI3K p85 and represses its levels, thus inhibiting the expression of the downstream molecules, p-Akt, p-mTOR and Snail, and ultimately leading to alleviation of EMT. Conversely, the cells treated with silica result in the enhanced expression of lncRNA MALAT1, which competitively binds to miR-503 and depresses its expression. When miR-503 is silenced, PI3K p85 bounding to miR-503 is released and thereby activates the downstream molecules, thus leading intensified process of EMT.

We have identified that the expression levels of miR-503 are significantly decreased in the tissues of silica-induced pulmonary fibrosis and two cell lines (HBE and A549) treated with silica compared with their control groups. Furthermore, miR-503 overexpression can inhibit EMT, slowing down the progression of pulmonary fibrosis. While accumulated evidence indicated that the expression of miR-503 varies in different organs and diseases. Some studies revealed that the expression of miR-503 is increased in several kinds of cancers^41–44^. *S Corbetta et al*.^41^ demonstrated that miR-503 is increased in parathyroid adenomas compared with normal parathyroid tissue. *Li et al*.^42^ suggested that the expression of miR-503 is up-regulated in the adenocarcinoma. Moreover, *Zhao et al*.^43^ revealed that the increase of miR-503 is associated with tumorigenesis of retinoblastoma. However, on the contrary, some other studies reported the declined expression of miR-503 in cervical cancer^19^, non-small cell lung cancer (NSCLC)^45^ and hepatocellular carcinoma(HCC)^46^. These data showed that the expression of miR-503 appears to be disease-specific or cell-type specific.

It is well characterized that miRNAs play its biological role mainly by binding to the 3′-UTR of the target genes. So we predicted the target genes by the bioinformatics tools and found that PI3K p85 is a target of miR-503. PI3K p85, also named PIK3R1, is a regulatory subunit of phosphatidylinositol 3-kinase (PI3K), and it also consists of a catalytic subunit, p110^47^. Moreover, *Robert J*. *Cain et al*.^48^ have already demonstrated the role of phosphatidylinositol 3-kinase in cell migration. In addition, *Andrei V*. *Bakin et al*.^49^ have found that the function of PI3K p85 is required for TGF-β-mediated EMT. In this study, we have identified that PI3K p85 is highly expressed in silica-induced lung fibrotic tissues, HBE cells, A549 cells, which is consistent with the negative expression of miR-503. While knockdown of PI3K p85 in HBE cells and A549 cells by siRNA can attenuate the process of EMT, thus alleviating the severity of fibrosis. One miRNA could have multiple target genes, while one gene can be targeted by several miRNAs. In present study, we have identified PI3K p85 is a target gene of miR-503, and a number of studies have demonstrated that PI3K p85 can be regulated by some other miRNAs. For example, *Li et al*.^50^ found that miR-21 could suppress tumor cell migration and invasion by reversing EMT via the PI3K/Akt pathway. And the research by *Wang et al*.^51^ also demonstrated that PIK3R1 is a target gene of miR-29b, which could prevent liver fibrosis by attenuating hepatic stellate cell activation and inducing apoptosis through PI3K/Akt signaling pathway. Emerging evidence implies that several miRNAs are anti-fibrotic modulators in the lung, such as miR-21, miR-29, and miR-200^12, 52^. These investigations suggest that numerous miRNAs could interact with the same or different genes thus forming a complex miRNA regulatory network in lung fibrotic diseases. Understanding of the signaling network is essential to explore the mechanisms and the new therapeutic targets of the pulmonary fibrotic diseases.

Recently, several scholars indicated that the contribution of epithelial cells to the population of fibroblasts is negligible by using lineage tracing in transgenic mice. However, *M*. *Angela Nieto et al*.^53^ demonstrated that the role of EMT in fibrosis can be explained by the partial EMT hypothesis. Partial EMT means cells are no longer thought to oscillate between the full epithelial and full mesenchymal states, but rather, they move through a spectrum of intermediary phases. This partial EMT hypothesis is also supported by *Marmai’s* research in lung fibrosis, in which a subset of epithelial cells of patients with IPF expresses both epithelial and mesenchymal markers^54^. Furthermore, *Wang et al*.^55^ also identified that MWCNTs could directly promote epithelial-mesenchymal transition and activate myofibroblasts both *in vivo* and *in vitro* in multiwall carbon nanotubes (MWCNTs)-induced pulmonary fibrosis. In order to testify whether silica could induce alveolar type II epithelial cell EMT, we also verified our previous experiments in the murine primary alveolar type II epithelial cells. After treated the cells with different concentrations of silica for 48 hours, we found the cells undergo morphological changes. And western blot analysis showed the decrease of E-cadherin protein expression and the increase of p-PI3K p85, PI3K p85, vimentin and α-SMA with the increase of the treatment concentration of silica. These results further support the hypothesis of partial EMT by using different types of cells, and *in vivo* study as well. Together with former studies, the present results may provide a new insight into EMT during pulmonary fibrosis.

The protein kinase Akt, downstream of PI3K, also known as PKB, regulates multiple biological functions such as growth, proliferation, migration and apoptosis^56, 57^. Having identified PI3K p85 is the target gene of miR-503, we were also wondering which signaling pathway is the key point in alleviating EMT in pulmonary fibrosis. Several researchers have reported the role of Akt in the biological process of EMT which supports our study. It has been reported that miR-21 suppresses breast cancer cell migration and reverses EMT through targeting PIK3R1 via PI3K/Akt signaling pathway^50^. *Yuan et al*.^58^ have also pointed out that p-Akt signaling regulates the EMT, tumor migration and invasion. In our study, the expression levels of p-Akt and mesenchymal markers are decreased after the knockdown of PI3K p85 in silica-treated HBE cells, which is consistent with these previous reports. In addition, the over expression of miR-503 could also suppress p-Akt expression both *in vivo* and *in vitro*, which all suggest that miR-503 limits the development of EMT via PI3K/Akt signaling pathway. mTOR (mammalian target of rapamycin) is a key kinase downstream of PI3K/Akt. Interestingly, PI3K/Akt/mTOR pathway has also emerged as a central signaling pathway involved in the regulation of EMT. *Lau and Leung* ^59^ have identified that PI3K/Akt/mTOR is required for insulin-like growth factor 1-induced E-cadherin down-regulation and up-regulation of E-cadherin transcriptional repressors, Snail and Slug, in human ovarian cancer cells. Snail is a transcription factor which has been recognized as the suppressor of E-cadherin and the inducer of EMT^60^. As Snail is an important negative regulator of E-cadherin, we detected that the expression of Snail is up-regulated in silica-treated lung tissues and HBE cells. Furthermore, over-expression of miR-503 and knockdown of PI3K p85 can down-regulate the expression of Snail, which confirms that miR-503 regulates EMT via the regulation of the transcription factor Snail.

Although we have demonstrated that the expression of miR-503 is down-regulated in silica-induced pulmonary fibrotic tissues and HBE cells, the mechanisms for the alterations of miR-503 are still obscure. *Zhou et al*.^23^ identified a potential epigenetic mechanism for the explanation of the down-regulation of miR-503 in HepG2 and LO2 cells. Two CpG enriched islands were found near the translational start site of miR-503. It means the expression of miR-503 could be possibly regulated by the modulation of methylation of the CpG islands. Furthermore, emerging evidence demonstrated that several miRNAs could be modulated by some long non-coding RNAs (lncRNAs), which work as the competing endogenous RNAs (molecular sponges) for the miRNAs, thus resulting in the down-regulation of miRNAs levels^61, 62^. Therefore, a critical issue for better understanding is that whether there are some lncRNAs that could sponge miR-503. With the help of several bioinformatics software, we found that lncRNA MALAT1 might be the potential target of miR-503. And then we confirmed that MALAT1 could bind to miR-503 directly by performing RNA pull-down assay and dual-luciferase reporter gene assay. Furthermore, knockdown of MALAT1 released miR-503 and inhibited the expression of its target gene PI3K p85 thus alleviating the process of EMT. LncRNA MALAT1 is a 8.7k-nt-long macromolecule which is highly conserved in mammals. The abundance of MALAT1 also provides great possibility to be a well-sponge platform for many kinds of miRNAs, not only miR-503. *Chou et al*.^63^ have reported that lncRNA MALAT1 could down-regulate the expression of miR-1 by acting as an endogenous competing RNA and induce migration and invasion of human breast cancer cells. To our knowledge, this is the first report showed miR-503 could be sponged by lncRNA MALAT1.

In summary, our present study has significant implications regarding the current understanding of the molecular mechanisms underlying the silica-induced lung fibrosis. It concentrated on the EMT-suppressive effects of miR-503 on the silica-induced pulmonary fibrosis via the classical PI3K/Akt/Snail signaling pathway, and the lncRNA MALAT1 serves as a molecular sponge to competitively decrease the expression of miR-503. Although our study results are not fully comprehensive, it may also provide research strategies for the molecular therapeutic target of pulmonary fibrosis to some extent.

## Materials and Methods

### Ethics statement

All animal studies were conducted according to humane animal care standards and were approved by the Ethics of Committee of Nanjing Medical University (Nanjing, China). And we confirm that all experiments were performed in accordance with relevant guidelines and regulations.

### Animal studies

Male C57BL/6 mice (4–6 weeks of age, 18–20 g) were purchased from Shanghai Laboratory Animal Center (SLAC, Shanghai, China), and were fed in sterile-free conditions of the animal center for one week before experiment to adapt to the environment. All animal studies were approved by the Ethics of Committee of Nanjing Medical University (Nanjing, China). Silica-induced mouse pulmonary fibrosis model was performed by intratracheal instillation of 50 μl 50 g/l silica turbid liquids for the three experimental group, and with equal amount of saline for the control group (n = 10 for each group). The mice were sacrificed on day 7, 14 and 28 after the treatment. The lungs were collected, quick-frozen in liquid nitrogen and store at −80 °C for further study. The miR-503 overexpression mouse lung fibrosis model was conducted by intratracheal instillation of 200 nmol/kg miR-503 agomir (RiboBio Co, Ltd, Guangzhou, China) after silica instillation. And on day 7, 14, 21 after the molding, 120nmol/kg miR-503 agomir were given to the miR-503 over expression group via tail vein injection. The mice were sacrificed at day 28.

### Cell culture and transfection

Human bronchial epithelial cells (HBE) and human lung adenocarcinoma A549 cells were obtained from American Type Culture Collection (ATCC, Manassas, USA) and were maintained at 37 °C with 5% CO_2_ in Dulbecco’s modified Eagle’s medium (DMEM, Life Technologies/Gibco, Grand Island, NY), supplemented with 10% fetal bovine serum (FBS, Life Technologies/Gibco, Grand Island, NY), 100 U/ml penicillin and 100 μg/ml streptomycin (Life Technologies/Gibco, Gaithersburg, MD).

Silica-induced HBE and A549 cells epithelial-mesenchymal transition model was conducted by adding silica suspension (prepared by mixing silica with DMEM) to the cells, and cultured in an incubator for 48 hours. Transfection of 50 nM miR-503 mimic (RiboBio Co, Ltd, Guangzhou, China) and PI3K p85 siRNA (RiboBio Co, Ltd, Guangzhou, China) were performed the day before the treatment of silica suspension following the manufacturer’s protocol.

### Tissue pathological sections

Lung tissues from each mouse were collected and were soaked in paraformaldehyde overnight. The tissues were embedded in paraffin and sectioned to 4 μm slices before stained with hematoxylin and eosin.

Sections were scanned by Pannoramic Scanning Electron Microscope and assessed the distribution range of fibrosis and severity of lesion. The structural changes of the lung tissues because of silica treatment were evaluated based on the degree of cellular proliferation, alveolar wall thickening, inflammatory lesions and collagen deposition or fibrosis. The alterations were graded from the aspects of severity and distributions as follows. The grading system was used for each group of animals^64^. For lesion distribution over the lung: 0 = absent, 1 = rare/occasional (10% of the lung area), 2 = sparse/limited (10–25% of the lung area), 3 = moderate (25–50% of the lung area), 4 = extensive (50–75% of the lung area), 5 = very extensive/predominant (over 75% of the lung area). For severity of lesions: o = nothing/zero, 1 = marginal, 2 = slight, 3 = moderate, 4 = severe, 5 = very severe.

### RNA extraction and qRT-PCR analysis

The total RNA of the cells and the lung tissues were extracted by using Trizol (Life Technologies/ambion, Carlsbad, CA) in accordance with the experimental instructions.

Thermo Nanodrop 2000 was used to detect the quality and the concentration of the extracted RNA. 500 ng total RNA was added to the reverse transcription reaction system (10 μl), and was reverse transcribed to cDNA by the specific RT primer according to the manufacturer’s instructions (TaKaRa Bio Inc, Japan). The miR-503 expression was performed using SYBR Green methods (TaKaRa Bio Inc, Japan) by qRT-PCR (ABI7900 real-time PCR instrument). The miR-503 expression levels were normalized to U6 (interval reference) and the lncRNA MALAT1 expression levels were normalized to GAPDH (interval reference) and calculated via 2^−ΔΔCt^ method.

### Western blot analysis

Expression of the target PI3Kp85 and EMT markers were determined by western blot analysis. The total protein of the tissues and the cells were extracted in lysis buffer (T-PER Tissue Protein Extraction Reagent and M-PER Mammalian Protein Extraction Reagent, Thermo Scientific Pierce) and 100 μg protein samples were separated by 12.5% SDS-PAGE and transferred to a PVDF membrane. The membrane was blocked in 5% defatted milk for more than one hour and incubated with the specific primary antibody at 4 °C overnight. After overnight incubation the membrane was probed with goat anti-rabbit or goat anti-mouse secondary antibody (1:1000, Beyotime, China) for one hour, and then, the membrane was exposed to the ChemiDoc XRS+ imaging system (Bio-Rad Laboratories, Inc).

Primary Antibodies: rabbit monoclonal antibody against PI3K p85 (#4257, 1:1000 dilution, Cell Signaling Technology), rabbit monoclonal antibody against p-PI3K p85 (#4228, 1:1000 dilution, Cell Signaling Technology), rabbit monoclonal antibody against α-SMA (ab124964, 1:1000 dilution, Abcam), rabbit monoclonal antibody against vimentin (#5741, 1:1000 dilution, Cell Signaling Technology), rabbit monoclonal antibody against E-cadherin (#3195, 1:1000 dilution, Cell Signaling Technology), rabbit monoclonal antibody against Akt (#4691, 1:1000 dilution, Cell Signaling Technology), rabbit monoclonal antibody against p-Akt (#4060, 1:2000 dilution, Cell Signaling Technology), rabbit monoclonal antibody against mTOR (#2983, 1:1000 dilution, Cell Signaling Technology), rabbit monoclonal antibody against p-mTOR (#5536, 1:1000 dilution, Cell Signaling Technology), rabbit monoclonal antibody against Snail (#3879, 1:1000 dilution, Cell Signaling Technology) and mouse monoclonal antibody against GAPDH (#5174, 1:1000 dilution, Cell Signaling Technology).

### Immunohistochemistry

Four-μm-thick formalin-fixed, paraffin-embedded serial sections of lung tissues were used for immunohistochemistry analysis of E-cadherin and vimentin expression. The sections were immersed in xylene for 15 minutes before being rehydrated in water by using an ethanol gradient. Then the sections were immersed in citric acid (pH 6.0; DAKO) for 10 minutes, after the samples were cooled to room temperature, the sections were washed with water and PBS buffer for 15 minutes before incubated with 3% H_2_O_2_ for 10 minutes. Then the sections were blocked with 5% BSA for 30 minutes before incubated with the primary antibody overnight at 4 °C. Color development was performed by using a DAB color development kit (DAKO). Sections were scanned by Pannoramic Scanning Electron Microscope to view the images.

### Immunofluorescence assay

HBE cells were cultured and plated in a small dish with a cover slip. The cells were treated with silica turbid liquid the day before fixed with 3% formaldehyde in PBS, and washed with PBS for 5 min, and then blocked with 5% BSA for 20 min. For α-SMA staining, cells were incubated with α-SMA primary antibody (ab124964, 1:200, Abcam) at 4 °C overnight. The next day, the cells were washed with PBS (three times, 5 min each), and then stained with the anti-rabbit secondary antibody 60 min at 37 °C and kept in a dark place. Finally, cells were washed with glycerol buffer and imaged with a fluoroscope (Olympus, Tokyo, Japan).

### RNA pull-down assay

HBE cells were cultured and total RNA was extracted after 48 h. 10 μl of the samples were reserved for input. MiR-503 (5 μl, 50 pmol) and negative control RNA (5 μl, 50 pmol) were labeled with desthiobiotin and connected to the streptavidin magnetic beads (Thermo Scientific). To ensure better binding, the beads were pre-washed with 0.1 M NaOH, 50 mM NaCl and 100 mM NaCl according to the manufacturer’s protocol. The remaining lysates were incubated with the streptavidin magnetic beads for 60 min at 4 °C with agitation or rotation. After binding process, the beads were washed twice with the 1× wash buffer (Thermo Scientific) and incubated with the elution buffer (Thermo Scientific) at 37 °C with agitation for 45 min. Then, collect the supernatant for qRT-PCR analysis.

### Plasmid constructs and dual luciferase reporter gene assays

We cloned sequences containing the binding region of miR-503 in PI3K p85 mRNA and lncRNA MALAT1 and their mutated version were cloned into the psiCHECK2 vector or pGL3-control vector (Generay Biotech Co., Ltd, Shanghai, China) at the 3′ region of the luciferase gene. And the PI3K p85 transcript sequence was synthesized and subcloned into pcDNA3.1 vector to construct PI3K p85 overexpression plasmid (Generay Biotech Co., Ltd, Shanghai, China) and was co-transfected with miR-503 mimic into HBE and A549 cells for the rescue experiment.

HBE or A549 cells were cultured in 24-well plates and transfected with 400 ng of either firefly luciferase reporter plasmids (pGL3-MALAT1-wt-3′-UTR; pGL3-MALAT1-mut-3′-UTR) together with 25 ng renilla luciferase construct (pRL-SV40), or 300ng of psiCHECK2-PIK3R1-wt-3′-UTR or psiCHECK2-PIK3R1- mut-3′-UTR combined with 50 nM miR-503 or miR-NC mimic using Reagent (RiboBio Co, Ltd, Guangzhou, China) according to the manufacturer’s protocol. Firefly and renilla luciferase activities were measured 48 hours after transfection using the Dual Luciferase Reporter Assay Kit (Promega) according to a protocol provided by the manufacturer.

### Statistical analysis

All experiments were repeated at least three times and the statistics were analysed by SPSS 20.0. The data were presented by means ± SD. Independent-samples *t* test was used for the analysis of two groups and one-way analysis of variance (ANOVA) for three or more groups with Dunnett’s test (none treatment as the control group). *P* value < 0.05 was considered statistically significant.

### Data Availability

All data generated or analysed during this study are included in this published article (and its Supplementary Information files).

## Electronic supplementary material

Supplementary file
