# Supplementary material for: MiR-503 modulates epithelial-mesenchymal transition in silica-induced pulmonary fibrosis by targeting PI3K p85 and is sponged by lncRNA MALAT1
Source: Sci Rep. 2017 Sep 12;7:11313. doi: 10.1038/s41598-017-11904-8 (PMC5596016; doi:10.1038/s41598-017-11904-8)
Supplement: Supplementary file 1 — Supplementary file [file 41598_2017_11904_MOESM1_ESM.pdf]

MiR-503 modulates epithelial-mesenchymal transition in silica-induced pulmonary fibrosis by targeting PI3K p85 and is sponged by lncRNA MALAT1

Weiwen Yan[1,†], Qiuyun Wu[1,2,†], Wenxi Yao[1], Yan Li[1], Yi Liu[1], Jiali Yuan[1], Ruhui Han[1], Jingjin Yang[1], Xiaoming Ji[1], Chunhui Ni[1,*]

[1] Department of Occupational Medicine and Environmental Health, Key Laboratory of Modern Toxicology of Ministry of Education, School of Public Health, Nanjing Medical University, Nanjing 211166, China.

[2] Department of Hygiene, School of Public Health, Xuzhou Medical University, Xuzhou 221004, China

† These authors contributed equally to this work.

[*] Corresponding authors

[*] Correspondence to: Department of Occupational Medicine and Environmental Health, Key Laboratory of Modern Toxicology of Ministry of Education, School of Public Health, Nanjing Medical University, Nanjing 211166, China. Tel: +86 25 86868418; Fax: +86 25 86868499; Email: chni@njmu.edu.cn, chninjmu@126.com

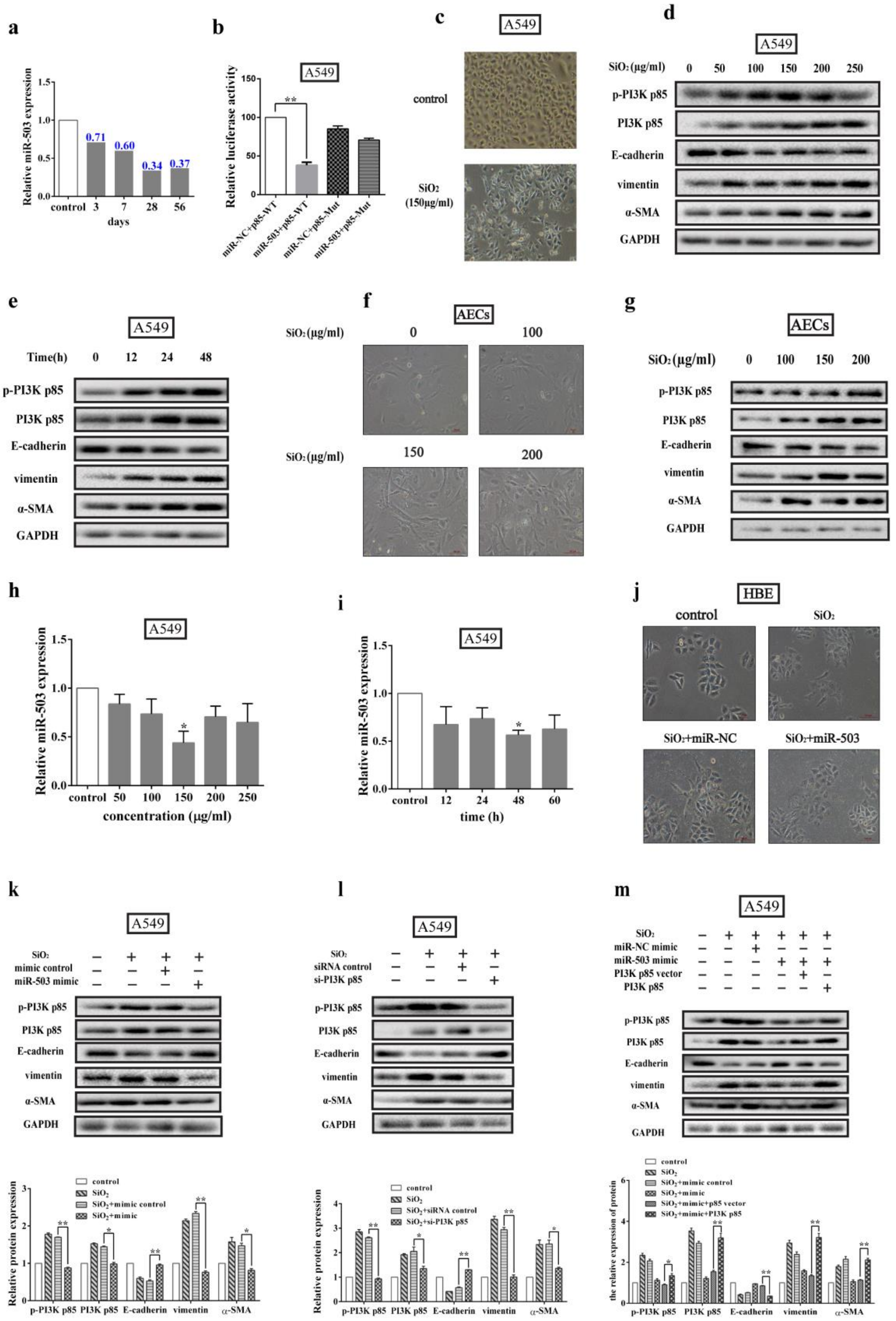
a
Relative miR-503 expression
control 3 7 28 56
days
0.71 0.60 0.34 0.37
b
A549
Relative luciferase activity
miR-NC+p85-WT
miR-503+p85-WT
miR-NC+p85-Mut
miR-503+p85-Mut
c
A549
control
SiO2 (150μg/ml)
d
A549
SiO2 (μg/ml) 0 50 100 150 200 250
p-PI3K p85
PI3K p85
E-cadherin
vimentin
α-SMA
GAPDH
e
A549
Time(h) 0 12 24 48
f
AECs
SiO2 (μg/ml) 0 100 150 200
g
AECs
SiO2 (μg/ml) 0 100 150 200
h
A549
Relative miR-503 expression
control 50 100 150 200 250
concentration (μg/ml)
i
A549
Relative miR-503 expression
control 12 24 48 60
time (h)
j
HBE
control
SiO2
SiO2+miR-NC
SiO2+miR-503
k
A549
SiO2
mimic control
miR-503 mimic
Relative protein expression
control
SiO2
SiO2+mimic control
SiO2+mimic
l
A549
SiO2
siRNA control
si-PI3K p85
Relative protein expression
control
SiO2
SiO2+siRNA control
SiO2+si-PI3K p85
m
A549
SiO2
miR-NC mimic
miR-503 mimic
PI3K p85 vector
PI3K p85
the relative expression of protein
control
SiO2
SiO2+mimic control
SiO2+mimic
SiO2+mimic+p85 vector
SiO2+mimic+PI3K p85

Supplementary Fig.1 (a) Relative expression levels of miR-503 in mouse lung tissues were determined by microarray analysis. (b) Effects of miR-503 mimics on PI3K p85 3'-UTR luciferase reporters in A549 cells. Luciferase activities were calculated as the ratio of firefly/renilla activities and normalized to the miR-NC+p85 WT group, $^{**}P<0.01$ versus the miR-NC+p85 WT group. (c) The morphological changes of A549 cells treated with 0 and 150μg/ml silica for 48 hours. The pictures were captured by the inverted microscope (Olympus).The scale bar is 100 μm. (d-e) The protein expression of p-PI3K p85, PI3K p85, vimentin and α-SMA significantly increased and the expression of E-cadherin significantly decreased when treated with different doses of silica for 48h and 150μg/ml silica for different time points in A549 cell lines. (f) The morphological changes of the murine alveolar type Ⅱ epithelial cells (AECs) treated with different concentrations of silica (0, 100, 150, 200μg/ml) for 48 hours. The pictures were captured by the inverted microscope (Olympus).The scale bar is 100 μm. (g) The protein expression of p-PI3K p85, PI3K p85, vimentin and α-SMA significantly increased and the expression of E-cadherin significantly decreased with the increase of the silica concentration in the murine alveolar type Ⅱ epithelial cells (AECs). (h-i) The miR-503 expression levels were significantly decreased in A549 cells treated with different doses of silica for 48h and 150μg/ml silica at different time points.$^{*}P<0.05$ versus the control group. (j) MiR-503 mimics could partly reverse the morphological changes of HBE cells compared with the SiO_2+miR-NC mimic group. (k) MiR-503 mimics reversed the protein expression levels of the target PI3K p85 and EMT markers (E-cadherin, vimentin and α-SMA) in A549 cells. And the relative protein expression levels were analyzed by the ImageJ program, $^{*}P<0.05$ and $^{**}P<0.01$ versus the SiO_2+miR-NC mimic group. (l) The siRNA of PI3K p85 reduced the protein expression of PI3K p85 and alleviated the process of epithelial mesenchymal transformation in A549 cells, $^{*}P<0.05$ and $^{**}P<0.01$ versus the SiO_2+siRNA control group. (m) Overexpression of PI3K p85 in A549 cells significantly restored the inhibitory effects of miR-503 in the process of EMT by rescue experiment. And the relative protein expression levels were analyzed by the ImageJ program, $^{**}P<0.05$ and $^{**}P<0.01$ versus the SiO_2+mimic+p85 vector group.

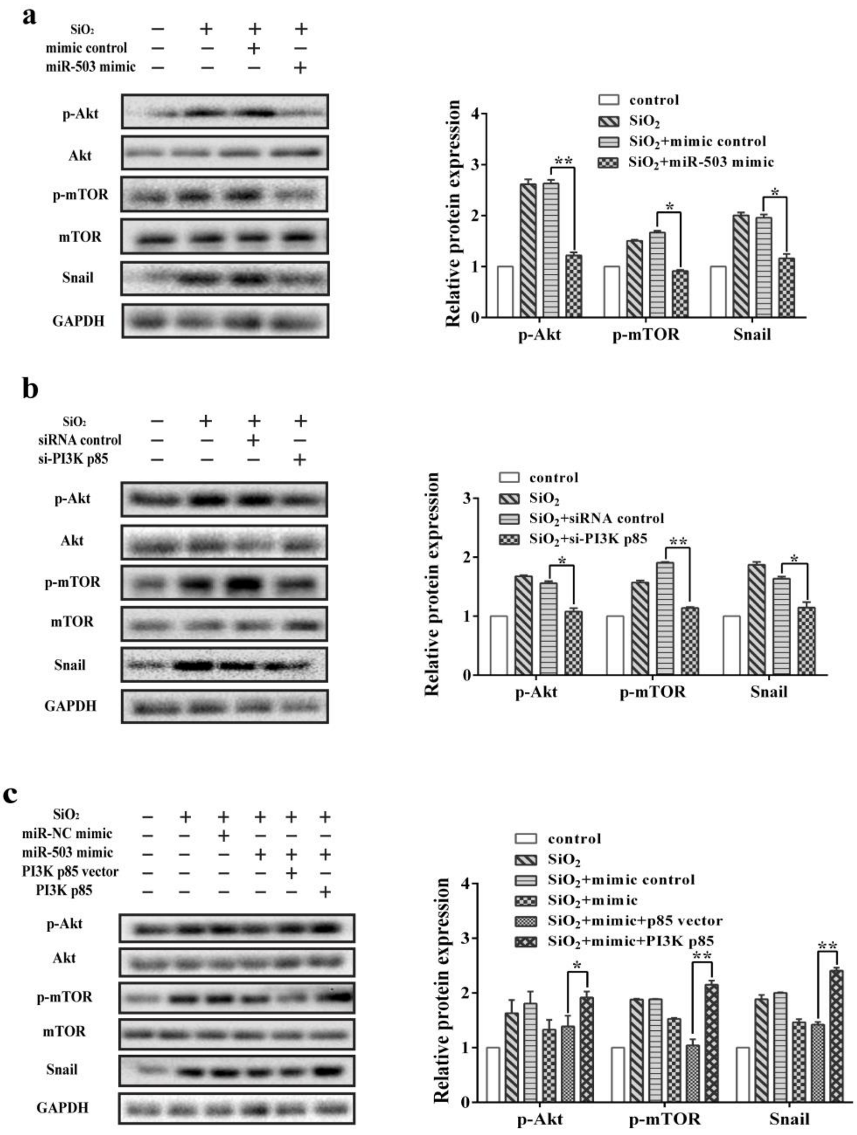

Supplementary Fig.2 miR-503 influences EMT through PI3K/Akt/mTOR/Snail signaling pathway in A549 cells. (a) miR-503 mimics significantly decreased the protein levels of p-Akt, p-mTOR and Snail in A549 cells. The relative protein levels were determined by the ImageJ program,,**P*<0.05 and ***P*<0.01 versus the control group. (b) The transfection of PI3K p85 siRNA together with the silica treatment in A549 cells reduced the expression of p-Akt, p-mTOR and Snail, **P*<0.05 and ***P*<0.01 versus the SiO_2+siRNA control group. (c) Co-transfection with PI3K p85 overexpression plasmid and miR-503 mimics in A549 cells restored the protein expression levels of p-Akt, p-mTOR and Snail which were inhibited by miR-503 mimics, **P*<0.05 and ***P*<0.01 versus the SiO_2+mimic+p85 vector group.

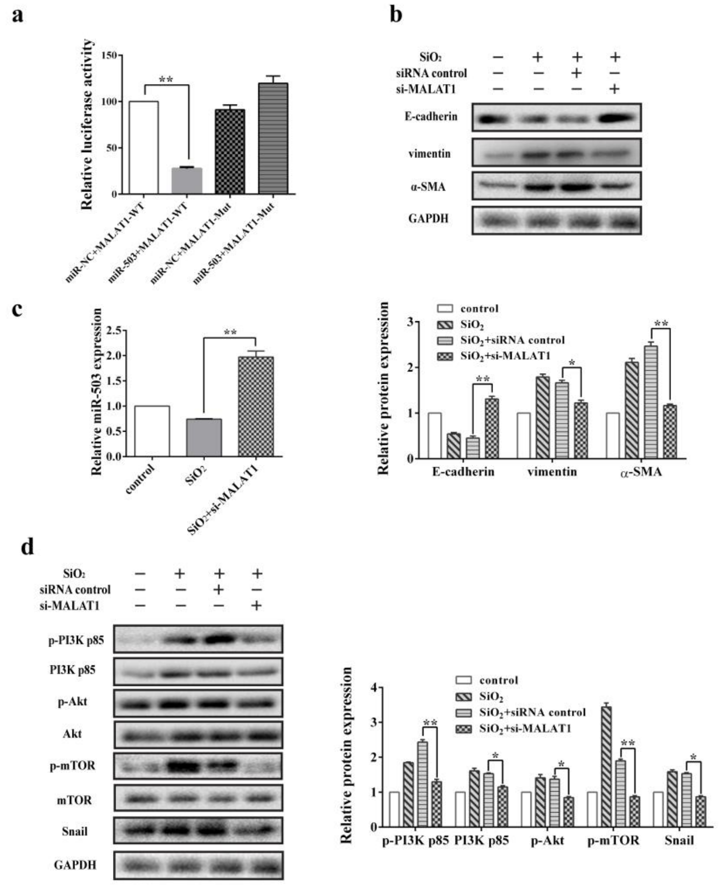

Supplementary Fig.3 LncRNA MALAT1 promotes EMT via binding to miR-503 directly in A549 cells. (a) The performance of the luciferase reporter gene assay in A549 cells identified the interaction between lncRNA MALAT1 and miR-503, luciferase activities were calculated as the ratio of firefly/renilla activities and normalized to the miR-NC+MALAT1 WT group,***P*<0.01 versus the miR-NC+MALAT1 WT group. (b) LncRNA MALAT1 siRNA significantly alleviated the process of EMT assessed by Western blot in A549 cells. The relative protein levels were determined by the ImageJ program,, **P*<0.05 and ***P*<0.01 versus the SiO_2+siRNA control group. (c) The level of miR-503 in A549 cells was significantly increased after knockdown of MALAT1 determined by qRT-PCR, ***P*<0.01 versus the SiO_2 group. (d) The protein levels of p-PI3K p85, PI3K p85, p-Akt, p-mTOR and Snail decreased after silencing of lncRNA MALAT1. The relative protein levels were determined by the ImageJ program,, **P*<0.05 and ***P*<0.01 versus the SiO_2+siRNA control group.
